# Synthesis and crystal structure of *rac*-2-(1,3-dioxo­isoindolin-2-yl)ethyl 4-methyl-*N*-phenyl-*N*′-(tri­iso­propyl­sil­yl)benzene­sulfondiimidoate: the first member of a new substance class

**DOI:** 10.1107/S2056989022005904

**Published:** 2022-06-10

**Authors:** Erik Friedrich, Timo Heinrich, Lara Rosenberger, Mireille Krier, Stephanie Marek, Michael Reggelin

**Affiliations:** a Alarich-Weiss-Str. 4, 64287 Darmstadt, Germany; b Merck KGaA, Frankfurter Str. 250, 64293 Darmstadt, Germany

**Keywords:** sulfondiimidoate, sulfondiimidate, crystal structure, bioisosters

## Abstract

The title compound {systematic name: *rac*-2-[7-methyl-4-(4-methylphenyl)-4-(phenylimino)-6,6-bis(propan-2-yl)-3-oxa-4λ^6^-thia-5-aza-6-silaoct-4-en-1-yl]-2,3-dihydro-1*H*-isoindole-1,3-dione}, C_32_H_41_N_3_O_3_SSi, was synthesized by desoxychlorination of 4-methyl-*N*-phenyl-*N*′-(triisopropyl­sil­yl)benzene­sul­fon­imid­am­ide and subsequent reaction with 2-(2-hy­droxy­eth­yl)isoindoline-1,3-dione. The racemic compound was crystallized from isopropanol. The structural characterization by single-crystal X-ray diffraction revealed two double-bonded nitro­gen atoms to the central sulfur atom and an overall crystal packing driven by its aromatic inter­actions.

## Abstract 2

The synthesis and crystal structure of *rac*-2-[7-methyl-4-(4-methyl­phen­yl)-4-(phenyl­imino)-6,6-bis­(propan-2-yl)-3-oxa-4λ^6^-thia-5-aza-6-silaoct-4-en-1-yl]-2,3-di­hydro-1*H*-iso­indole-1,3-dione are reported.

## 1. Chemical context

Since 2013 (Lücking, 2013 ▸, 2019 ▸), there has been an increased research inter­est in bioisosters of sulfonamides and sulfones. In addition to vigorous inter­est in the development of new synthetic procedures towards sulfonimidamides (Nandi & Arvidsson, 2018 ▸; Chen & Gibson, 2015 ▸; Wen *et al.*, 2016 ▸; Izzo *et al.*, 2017 ▸; Greed *et al.*, 2020 ▸; Liu *et al.*, 2021 ▸), activities towards the synthesis of sulfondi­imides have recently just begun (Zhang *et al.*, 2019 ▸; Bohmann *et al.*, 2019 ▸). With the synthesis of stable sulfondiimidamides, Zhang & Willis (2022 ▸) introduced a new functional group for medicinal chemistry.

The different aza-analogs of sulfonamides and sulfones have inter­esting properties for medicinal chemistry due to the (additional) nitro­gen atom(s). Besides the potential centrochirality of sulfur, the nitro­gen substituents offer new possibilities for functionalization optimizing steric demand, solubility and reactivity. 
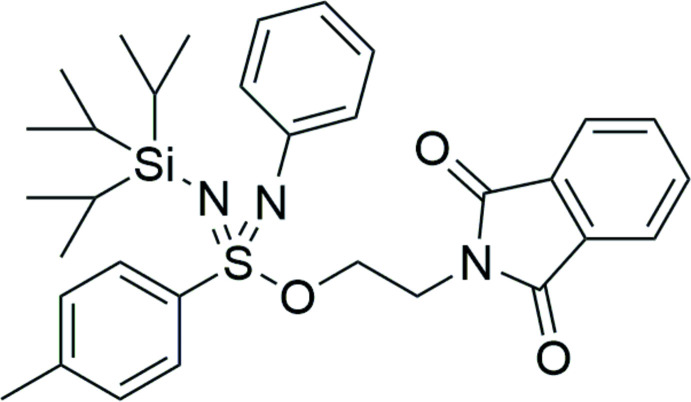


The herein reported sulfondiimidoate **1** is, based on extensive database searches, not yet described in the literature and therefore represents the first member of a new substance class. It can be described as an aza-oxo-inverse sulfonamide or an aza-analogue of a sulfonimidoate.

## 2. Structural commentary

**Figure 1:**
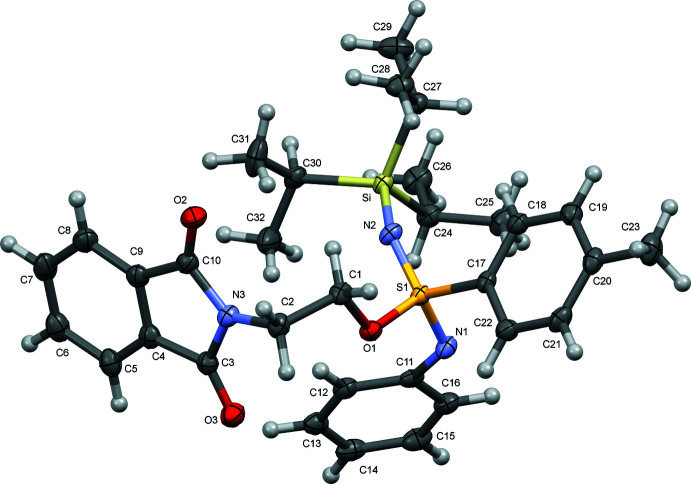
The mol­ecular structure of 2-(1,3-dioxoisoindolin-2-yl)ethyl-4-methyl-*N*-phenyl-*N′*-(triisopropyl­sil­yl)benzene­sulfondiimidoate (**1**) with the atomic numbering scheme. Displacement ellipsoids are drawn at the 50% probability level.

The title compound **1** crystallizes in the triclinic crystal system and *P* 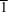 as the centrosymmetric space group, having one mol­ecule in the asymmetric unit (Fig. 1 ▸). Geometric parameters may be regarded as normal. A selection is listed in Table 1 ▸.

The tetra­hedral mol­ecular structure shows a sulfur as the central atom, surrounded by four substituents, including two sulfur–nitro­gen double bonds. As a result of the steric repulsion of the aniline ring and the bulky triisopropyl­silyl group, the angle N2—S1—N1 at 126.60 (9)° is larger than the typical tetra­hedral angle (109.5°), whereas the angle between the aniline and toluene ring (N1—S1—C17) and also the 1,3-dioxoisoindolin moiety (N1—S1—O1) are smaller at 101.98 (9) and 105.93 (8)°, respectively. The remaining angle (N2—S1—O1) is 107.27 (8)°. The bond lengths between S1—N1 [1.5139 (16) Å] and S1—N2 [1.4838 (16) Å] are similar to those observed in crystal structures of sulfoximines [1.484 Å; CSD refcode: LISJAZ (Lemasson *et al.*, 2007 ▸) or 1.518 Å; CSD refcode: NADNAH; (Mash *et al.*, 1996 ▸)], and therefore confirming the presence of the double bonds (Reggelin & Zur, 2000 ▸). The ring systems are planar (r.m.s values of 0.003 and 0.007 Å for the phenyl rings and 0.022 Å for the phthalimide).

## 3. Supra­molecular features

**Figure 2:**
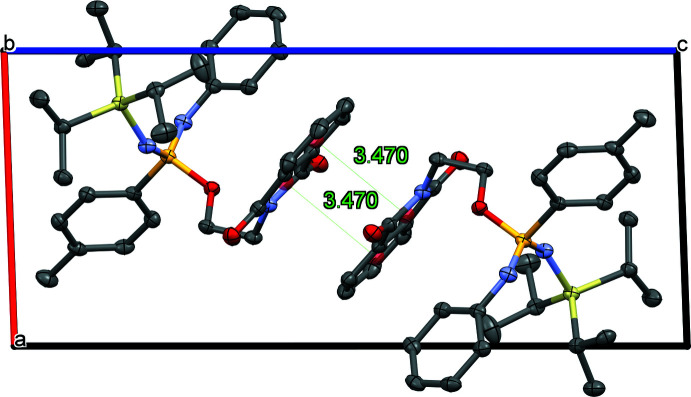
Crystal packing in *rac*-2-(1,3-dioxoisoindolin-2-yl)ethyl-4-methyl-*N*-phenyl-*N*′-(triisopropyl­sil­yl)benzene­sulfondiimidoate (**1**) viewed along the *b* axis. Anti­parallel stacking of the phthalimide occurs with a centroid–centroid distance of 3.470 (1) Å. Displacement ellipsoids are drawn at the 50% probability level and H atoms are omitted for clarity.

The title compound **1** contains secondary nitro­gen groups and a dicarboximide, which are hydrogen-bond acceptors, but no strong or moderate inter­molecular hydrogen bonds were detected in the crystal. Geometric details of some possible weak hydrogen bonds are listed in Table 2 ▸. This includes three borderline C—H⋯O hydrogen bonds, which link the chains *via* the operators 1 + *x*, −1 + *y*, *z* and 2 − *x*, 1 − *y*, 1 − *z*. The contact C31—H31⋯N1, involving a tertiary methyl group, connects the mol­ecules *via* the operator *x*, 1 + *y*, *z*. Fig. 2 ▸ shows the unit cell of the compound along the *b*-axis. It appears that the crystal structure contains anti-parallel π stacking inter­actions of the phthalimide between its electron-rich six-membered ring and electron-poor five-membered ring (Ahmed *et al.*, 2019 ▸). The centroid-to-centroid distance of 3.470 (1) Å, which is in the range of π–π stacking inter­actions, confirms its presence. The crystal packing is mainly driven by its attractive inter­molecular aromatic inter­actions, as can be shown by the Aromatics Analyser (feature available in *Mercury* as part of the CSD-Materials and CSD-Enterprise suites). The distance between centroids for which the assessment was labelled ‘strong’ equals to 4.11 Å (score: 9.3) and for the ‘moderate’ ones between 4.48 and 6.39 Å (score: 6.9–3.7) by the CCDC’s Aromatics Analyser using a score from 0 (no stabilizing contribution) to 10 (an ideal aromatic inter­action geometry) (assessment: ‘weak’ 0–3, ‘moderate’ 3–7, ‘strong’ 7–10. *Mercury* 2021.3.0 (Build 333817) used (Macrae *et al.*, 2020 ▸).

## 4. Database survey

The herein reported sulfondiimidoate **1** is, based on extensive database searches, not yet described in the literature. A Scifinder^n^ structure search with undefined bonds on all substituents of the sulfur and a substituent on the oxygen atom resulted in no structure matches as drawn (SciFinder; Chemical Abstracts Service: Columbus, OH; https://scifinder.cas.org; accessed: 06.05.2022). A broadly defined Cambridge Structural Database search with the five central atoms and any type of bonds (SMARTS pattern [#7]∼[#16](∼[#8])(∼[#6])∼[#7]) on CSD version 5.43 November 2021 plus update of March 2022 found 85 hits (Groom *et al.*, 2016 ▸), all of which are sulfonimidamides.

Restricting this query to a single bond (instead of any bond) between the sulfur and the oxygen returns zero hits. The mean distance between sulfur and oxygen in the 85 hits dataset is 1.436 with a standard deviation of 0.014. The distance S1—O1 (see also Table 1 ▸) is hence clearly a single bond and similar functional groups have not been missed by setting the query in too narrow a way.

## 5. Synthesis and crystallization

**Figure 3:**
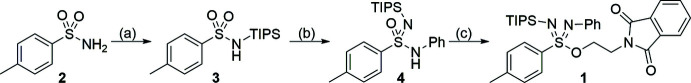
Synthesis of the sulfondiimidoate **1**. (*a*) TIPS-Cl, NEt_3_; (*b)* C_2_Cl_6_, PPh_3_, NEt_3_, aniline; (*c*) C_2_Cl_6_, PPh_3_, NEt_3_, *N*-hy­droxy­ethyl­phthalimide.

Mol­ecular schemes with the atom numbering used in the NMR assignments can be found in Figures S1–S3 in the supporting information. Solvent residue signals were used as inter­nal standard according to the literature [^1^H-NMR: δ (CHCl_3_) = 7.26 ppm; ^13^C-NMR: δ (CDCl_3_) = 77.16 ppm; (Gottlieb *et al.*, 1997 ▸)]. The synthesis is shown in Fig. 3 ▸.

***N*** **-(Tri** ***-iso*** **-propyl­sil­yl)-4-methyl­benzene­sulfonamide (3)**

7.51 mL (6.82 g, 35.0 mmol, 1.2 eq.) of TIPS-Cl and 12.1 mL (8.87 g, 87.6 mmol, 3.0 eq.) of NEt_3_ were added to a suspension of 5.00 g (29.2 mmol, 1.0 eq.) of *p*-toluene­sulfonamide (**2**) in 100 mL of CH_2_Cl_2_. After stirring for 62 h, 100 mL of 1*M* HCl were added to the reaction mixture. The aqueous layer was extracted with CH_2_Cl_2_ three times, the combined organic layers were dried over MgSO_4_, the solvent was removed under reduced pressure and the crude product was dissolved in 100 mL of CH_2_Cl_2_. After addition of 300 mL of petroleum ether, the CH_2_Cl_2_ was removed under reduced pressure. The resulting precipitate was filtered off and washed with pentane. After drying, the protected sulfonamide **3** (9.12 g, 27.8 mmol, 95%) was obtained as a colorless solid. *R_f_* 0.75 (20% EtOAc in penta­ne). M.p. = 427 K. IR (ATR)/cm^−1^ 1462, 1344, 1286, 1154, 1094, 1004, 936. ^1^H-NMR (CDCl_3_, 500 MHz, 300 K): δ = 7.80 (*d*, *J* = 8.3 Hz, 4-H_2_), 7.27 (*d*, *J* = 8.3 Hz, 3-H_2_), 4.43 (*bs*, 6-H_1_), 2.42 (*s*, 1-H_3_), 1.29 (*hep*., *J* = 7.5 Hz, 7-H_3_), 1.15 (*d*, *J* = 7.5 Hz, 8-H_18_) ppm. ^13^C-NMR (CDCl_3_, 125 MHz, 300 K): δ = 142.6 (2-C), 141.1 (5-C), 129.5 (3-C_2_), 126.2 (4-C_2_), 21.6 (1-C), 18.1 (8-C_6_), 12.1 (7-C_3_) ppm. Calculated for C_16_H_29_NO_2_SSi: C 58.67, H 8.92, N 4.28; found: C 58.68, H 9.30, N 4.53. ESI–MS: *m*/*z* = 328.18 [*M* + H]^+^, 677.33 [2*M* + Na]^+^.

**4-Methyl-** ***N*** **-phenyl-** ***N*** ′**-(tri-** ***iso*** **-propyl­sil­yl)benzene­sulf­on­im­id­amide (4)**

3.98 g (16.8 mmol, 1.1 eq) of C_2_Cl_6_ and 4.40 g (16.8 mmol, 1.1 eq) of PPh_3_ were heated to reflux of the solvent in 60 mL of CHCl_3_ for 6 h. After cooling to room temperature, 3.19 mL (2.32 g, 22.9 mmol, 1.5 eq) of NEt_3_ was added *via* syringe. After five minutes, the reaction mixture was cooled to 273 K. After another five minutes, 5.00 g (15.3 mmol, 1.0 eq) of 4-methyl-*N*-(triisopropyl­sil­yl)benzene­sulfon­amide (**3**) were added. After ten more minutes, 5.58 mL (5.69 g, 61.1 mmol, 4.0 eq) of aniline were added *via* syringe and the mixture was stirred for one h, at which point the reaction was stopped by the addition of 100 mL of saturated NH_4_Cl solution. The aqueous phase was extracted three times with 50 mL of CH_2_Cl_2_. The combined organic layers were dried over MgSO_4_, the solvent was removed under reduced pressure and the crude product was purified by flash chromatography (5% EtOAc in penta­ne) affording the sulfonimidamide **4** (5.64 g, 14.0 mmol, 92%) as a colorless solid. *R_f_* 0.63 (20% EtOAc in penta­ne). M.p. = 364 K. IR (ATR)/cm^−1^ 3228, 1600, 1480, 1410, 1347, 1282, 1141, 1091, 895. ^1^H-NMR (CDCl_3_, 500 MHz, 300 K): δ = 7.68 (*d*, *J* = 8.3Hz, 4-H_2_), 7.19–7.13 (*m*, 3/8-H_4_), 7.03–6.97 (*m*, 9/10-H_3_), 6.30 (*bs*, 6-H), 2.34 (*s*, 1-H_3_), 1.18–1.03 (*m*, 11/12-H_21_) ppm. ^13^C-NMR (CDCl_3_, 125 MHz, 300 K): δ = 142.2 (7-C), 141.0 (2-C), 138.9 (5-C), 129.2 (9-C_2_), 129.0 (3-C_2_), 127.1 (4-C_2_), 124.2 (8-C_2_), 121.2 (10-C), 21.5 (1-C), 18.5 (12-C_6_), 13.3 (11-C_3_) ppm. Calculated for C_22_H_34_N_2_OSSi: C 65.62, H 6.96, N 8.51; found: C 65.65, H 6.97, N 8.55. ESI–MS: *m*/*z* = 403.22 [*M* + H]^+^.

***rac*** **-2-(1,3-Dioxo-** ***iso*** **-indolin-2-yl)ethyl-4-methyl-** ***N*** **-phenyl-** ***N*** ′**-(tri-** ***iso*** **-propyl­sil­yl)­benzene­sulfondiimidoate (1)**

282 mg (1.19 mmol, 1.2 eq) of C_2_Cl_6_ and 313 mg (1.19 mmol, 1.2 eq) of PPh_3_ were heated to reflux of the solvent in 5 mL of CHCl_3_ for 6 h. After cooling to room temperature, 0.83 mL (603 mg, 5.96 mmol, 6.0 eq) of NEt_3_ were added *via* syringe. After five minutes, the reaction mixture was cooled to 273 K. After five more minutes, 400 mg (0.99 mmol, 1.0 eq) of 4-methyl-*N*-phenyl-*N′*-(triisopropyl­sil­yl)benzene­sulfonimid­amide (**4**) were added and the reaction mixture was stirred for 20 more minutes at 273 K, at which point 1.52 g (7.95 mmol, 8.0 eq) of 2-(2-hy­droxy­eth­yl)isoindoline-1,3-dione were added. The mixture was stirred for another 30 min and then quenched with 20 mL of saturated NH_4_Cl solution. After phase separation, the aqueous solution was extracted three times with 20 mL of CH_2_Cl_2_, the combined organic layers were dried over MgSO_4_, the solvent was removed under reduced pressure and the resulting crude product was purified by flash chromatography (8% EtOAc in penta­ne) affording the sulfondiimidoate **1** (447 mg, 0.78 mmol, 78%) as a colorless solid. Crystals suitable for X-ray structure analysis were obtained by recrystallization from *iso*-propanol. *R_f_* 0.16 (10% EtOAc in penta­ne). M.p. = 380 K. IR (ATR)/cm^−1^ 2941, 2862, 1712, 1594, 1488, 1391, 1294, 1056, 995. ^1^H-NMR (CDCl_3_, 500 MHz, 300 K): δ = 7.83–7.77 (*m*, 4/16-H_4_), 7.75–7.70 (*m*, 17-H_2_), 7.11–7.04 (*m*, 3/10-H_4_), 6.98–6.96 (*m*, 9-H_2_), 6.82 (*t*, *J* = 7.3 Hz, 11-H), 4.19–4.06 (*m*, 12-H_2_), 3.88 (*t*, *J* = 5.6 Hz, 13-H_2_), 2.30 (*s*, 1-H_3_), 0.94–0.88 (*m*, 6/7-H_21_) ppm. ^13^C-NMR (CDCl_3_, 125 MHz, 300 K): δ = 167.9 (14-C_2_), 144.6 (8-C), 142.4 (2-C), 139.3 (5-C), 134.0 (17-C_2_), 132.2 (1-C_2_), 129.3 (3-C_2_), 128.7 (10-C_2_), 127.5 (4-C_2_), 123.7 (9-C_2_), 123.4 (16-C_2_), 121.2 (11-C), 64.5 (12-C), 37.2 (13-C), 21.6 (1-C), 18.3 (7-C_3_), 18.3 (7′-C_3_), 13.3 (6-C_3_) ppm. Calculated for C_32_H_41_N_3_O_3_SSi: C 66.75, H 7.18, N 7.30; found: C 66.62, H 6.86, N 7.13. ESI–MS: *m*/*z* = 576.27 [*M* + H]^+^.

## 6. Refinement

Crystal data, data collection and structure refinement details are summarized in Table 3 ▸. Hydrogen atoms were refined isotropically using a riding model. The C—H bond distances were constrained to 0.95 Å for aromatic C—H moieties, and to 1.00, 0.99 and 0.98 Å for aliphatic C—H, CH_2_ and CH_3_ moieties, respectively. Methyl-H atoms were allowed to rotate but not to tip to best fit the experimental electron density. *U*_iso_(H) values were set to a multiple of *U*_eq_(C) with 1.5 for CH_3_, and 1.2 for C—H, CH_2_ groups, respectively.

## Supplementary Material

Crystal structure: contains datablock(s) I. DOI: 10.1107/S2056989022005904/zl5029sup1.cif

Structure factors: contains datablock(s) I. DOI: 10.1107/S2056989022005904/zl5029Isup2.hkl

Supporting information file. DOI: 10.1107/S2056989022005904/zl5029Isup4.cdx

Supporting information file. DOI: 10.1107/S2056989022005904/zl5029Isup5.cdx

Supporting information file. DOI: 10.1107/S2056989022005904/zl5029Isup6.cdx

Molecular schemes with atom numbering used in the NMR assignments. DOI: 10.1107/S2056989022005904/zl5029sup3.pdf

Supporting information file. DOI: 10.1107/S2056989022005904/zl5029Isup7.cml

CCDC reference: 2163661

Additional supporting information:  crystallographic information; 3D view; checkCIF report

## Figures and Tables

**Table 1:** Selected geometric parameters (Å, °)

S1—N1	1.5139 (16)	S1—C17	1.7718 (19)
S1—N2	1.4838 (16)	Si—N2	1.7240 (17)
S1—O1	1.6257 (14)	N1—C11	1.412 (2)
			
N1—S1—O1	105.93 (8)	N2—S1—N1	126.60 (9)
N1—S1—C17	101.98 (9)	S1—N2—Si	142.24 (11)
N2—S1—O1	107.27 (8)	N2—Si—C27	105.99 (9)

**Table 2:** Hydrogen-bond geometry (Å, °)

*D*—H⋯*A*	*D*—H	H⋯*A*	*D*⋯*A*	*D*—H⋯*A*
C15—H15⋯O2^i^	0.95	2.52	3.429 (3)	160
C22—H22⋯O2^ii^	0.95	2.64	3.348 (3)	132
C14—H14⋯O3^iii^	0.95	2.52	3.429 (3)	161
C31—H31*B*⋯N1^iv^	0.98	2.60	3.361 (3)	135

Symmetry codes: (i) 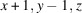 ; (ii) 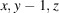 ; (iii) 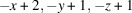 ; (iv) 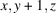 .

**Table 3:** Experimental details

Crystal data	
Chemical formula	C_32_H_41_N_3_O_3_SSi
*M*_r_	575.83
Crystal system, space group	Triclinic, *P* 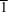
Temperature (K)	100
*a*, *b*, *c* (Å)	8.6752 (2), 8.8765 (2), 20.2299 (6)
α, β, γ (°)	78.107 (2), 87.922 (2), 89.512 (2)
*V* (Å^3^)	1523.37 (7)
*Z*	2
Radiation type	Cu *K*α
μ (mm^−1^)	1.61
Crystal size (mm)	0.21 × 0.16 × 0.06

Data collection	
Diffractometer	XtaLAB Synergy R, HyPix-Arc 150
Absorption correction	Gaussian (*CrysAlis PRO*; Rigaku, 2021 ▸)
*T*_min_, *T*_max_	0.555, 1.000
No. of measured, independent and observed [*I* > 2σ(*I*)] reflections	28106, 5410, 4426
*R*_int_	0.047
(sin θ/λ)_max_ (Å^−1^)	0.597

Refinement	
*R*[*F* ^2^ > 2σ(*F* ^2^)], *wR*(*F* ^2^), *S*	0.041, 0.108, 1.05
No. of reflections	5410
No. of parameters	368
H-atom treatment	H-atom parameters constrained
Δρ_max_, Δρ_min_ (e Å^−3^)	0.56, −0.38

Computer programs: *CrysAlis PRO* (Rigaku, 2021 ▸), *SHELXT2014/5* (Sheldrick, 2015*a* ▸), *SHELXL2018/1* (Sheldrick, 2015*b* ▸), *Mercury* (Macrae *et al.*, 2020 ▸) and *OLEX2* (Dolomanov *et al.*, 2009 ▸).

## supplementary crystallographic information

### Crystal data

**Table:**

C_32_H_41_N_3_O_3_SSi	*Z* = 2
*M**_r_* = 575.83	*F*(000) = 616
Triclinic, *P*1	*D*_x_ = 1.255 Mg m^−^^3^
*a* = 8.6752 (2) Å	Cu *K*α radiation, λ = 1.54184 Å
*b* = 8.8765 (2) Å	Cell parameters from 4930 reflections
*c* = 20.2299 (6) Å	θ = 4.5–72.1°
α = 78.107 (2)°	µ = 1.61 mm^−^^1^
β = 87.922 (2)°	*T* = 100 K
γ = 89.512 (2)°	Needle, colourless
*V* = 1523.37 (7) Å^3^	0.21 × 0.16 × 0.06 mm

### Data collection

**Table:**

XtaLAB Synergy R, HyPix-Arc 150 diffractometer	5410 independent reflections
Radiation source: Rotating-anode X-ray tube, PhotonJet R (Cu) X-ray Source	4426 reflections with *I* > 2σ(*I*)
Mirror monochromator	*R*_int_ = 0.047
Detector resolution: 10.0000 pixels mm^-1^	θ_max_ = 67.1°, θ_min_ = 4.5°
ω scans	*h* = −10→9
Absorption correction: gaussian (CrysAlisPro; Rigaku, 2021)	*k* = −10→10
*T*_min_ = 0.555, *T*_max_ = 1.000	*l* = −24→24
28106 measured reflections	

### Refinement

**Table:**

Refinement on *F*^2^	Primary atom site location: dual
Least-squares matrix: full	Hydrogen site location: inferred from neighbouring sites
*R*[*F*^2^ > 2σ(*F*^2^)] = 0.041	H-atom parameters constrained
*wR*(*F*^2^) = 0.108	*w* = 1/[σ^2^(*F*_o_^2^) + (0.0575*P*)^2^ + 0.4269*P*] where *P* = (*F*_o_^2^ + 2*F*_c_^2^)/3
*S* = 1.05	(Δ/σ)_max_ = 0.001
5410 reflections	Δρ_max_ = 0.56 e Å^−^^3^
368 parameters	Δρ_min_ = −0.38 e Å^−^^3^
0 restraints	

### Special details

**Table:**

Geometry. All esds (except the esd in the dihedral angle between two l.s. planes) are estimated using the full covariance matrix. The cell esds are taken into account individually in the estimation of esds in distances, angles and torsion angles; correlations between esds in cell parameters are only used when they are defined by crystal symmetry. An approximate (isotropic) treatment of cell esds is used for estimating esds involving l.s. planes.

### Fractional atomic coordinates and isotropic or equivalent isotropic displacement parameters (Å^2^)

**Table:**

	*x*	*y*	*z*	*U*_iso_*/*U*_eq_	
S1	0.64242 (5)	0.36921 (5)	0.76180 (2)	0.01884 (13)	
Si	0.82253 (6)	0.58177 (6)	0.83046 (3)	0.01969 (14)	
O1	0.53274 (15)	0.43170 (15)	0.69843 (7)	0.0213 (3)	
O2	0.37133 (16)	0.92827 (16)	0.67134 (7)	0.0286 (3)	
O3	0.61871 (16)	0.63745 (16)	0.53826 (7)	0.0293 (3)	
N1	0.75807 (18)	0.26207 (18)	0.73756 (9)	0.0224 (4)	
N3	0.47858 (18)	0.75010 (18)	0.61476 (8)	0.0220 (4)	
N2	0.67766 (18)	0.50250 (18)	0.79249 (8)	0.0215 (4)	
C17	0.5169 (2)	0.2380 (2)	0.81561 (10)	0.0191 (4)	
C11	0.8812 (2)	0.3081 (2)	0.69029 (10)	0.0215 (4)	
C4	0.6598 (2)	0.9038 (2)	0.54781 (10)	0.0238 (4)	
C30	0.8671 (2)	0.7773 (2)	0.77709 (11)	0.0249 (5)	
H30	0.941234	0.829050	0.801923	0.030*	
C16	1.0089 (2)	0.2104 (2)	0.69474 (11)	0.0252 (5)	
H16	1.013655	0.123095	0.730762	0.030*	
C9	0.5850 (2)	0.9926 (2)	0.58821 (10)	0.0247 (4)	
C20	0.3301 (2)	0.0244 (2)	0.90149 (11)	0.0252 (5)	
C18	0.5129 (2)	0.2342 (2)	0.88389 (10)	0.0227 (4)	
H18	0.573253	0.304358	0.901627	0.027*	
C3	0.5901 (2)	0.7473 (2)	0.56355 (10)	0.0226 (4)	
C21	0.3360 (2)	0.0304 (2)	0.83209 (11)	0.0268 (5)	
H21	0.275146	−0.039010	0.814108	0.032*	
C10	0.4654 (2)	0.8955 (2)	0.63090 (10)	0.0234 (4)	
C12	0.8784 (2)	0.4379 (2)	0.63815 (10)	0.0239 (4)	
H12	0.792938	0.506435	0.634980	0.029*	
C22	0.4293 (2)	0.1361 (2)	0.78878 (11)	0.0246 (4)	
H22	0.433214	0.138741	0.741556	0.030*	
C1	0.4033 (2)	0.5309 (2)	0.70767 (10)	0.0231 (4)	
H1A	0.429808	0.598387	0.738910	0.028*	
H1B	0.312386	0.468228	0.727047	0.028*	
C19	0.4200 (2)	0.1271 (2)	0.92686 (11)	0.0256 (5)	
H19	0.417784	0.123993	0.974110	0.031*	
C24	0.9990 (2)	0.4556 (2)	0.83641 (11)	0.0243 (4)	
H24	1.024122	0.441481	0.789435	0.029*	
C2	0.3681 (2)	0.6264 (2)	0.63933 (11)	0.0239 (4)	
H2A	0.366244	0.558188	0.606327	0.029*	
H2B	0.263929	0.671795	0.641762	0.029*	
C13	0.9999 (2)	0.4674 (2)	0.59088 (11)	0.0278 (5)	
H13	0.997509	0.556827	0.555804	0.033*	
C27	0.7421 (2)	0.5970 (2)	0.91717 (11)	0.0246 (4)	
H27	0.750357	0.492007	0.946548	0.030*	
C8	0.6216 (3)	1.1462 (2)	0.58321 (11)	0.0317 (5)	
H8	0.570588	1.207038	0.610828	0.038*	
C28	0.5708 (2)	0.6429 (3)	0.91904 (12)	0.0294 (5)	
H28A	0.558879	0.749867	0.894987	0.044*	
H28B	0.533930	0.632856	0.966109	0.044*	
H28C	0.510518	0.575262	0.897214	0.044*	
C25	0.9721 (2)	0.2933 (2)	0.87942 (12)	0.0296 (5)	
H25A	0.947197	0.300481	0.926309	0.044*	
H25B	1.065707	0.231402	0.877778	0.044*	
H25C	0.886315	0.244644	0.861586	0.044*	
C5	0.7750 (2)	0.9636 (2)	0.50117 (11)	0.0282 (5)	
H5	0.826384	0.902115	0.473913	0.034*	
C15	1.1291 (2)	0.2406 (3)	0.64663 (12)	0.0301 (5)	
H15	1.215207	0.172932	0.649736	0.036*	
C14	1.1246 (2)	0.3683 (3)	0.59417 (12)	0.0303 (5)	
H14	1.206057	0.387532	0.560883	0.036*	
C7	0.7370 (3)	1.2071 (3)	0.53569 (11)	0.0344 (5)	
H7	0.764486	1.312400	0.530483	0.041*	
C23	0.2284 (3)	−0.0901 (3)	0.94846 (12)	0.0357 (6)	
H23A	0.120046	−0.067014	0.938481	0.054*	
H23B	0.246165	−0.083527	0.995349	0.054*	
H23C	0.252958	−0.194293	0.942108	0.054*	
C26	1.1435 (2)	0.5273 (3)	0.85938 (12)	0.0333 (5)	
H26A	1.161285	0.629633	0.830942	0.050*	
H26B	1.233053	0.461466	0.855196	0.050*	
H26C	1.128079	0.536457	0.906621	0.050*	
C29	0.8374 (3)	0.7056 (3)	0.94995 (12)	0.0371 (6)	
H29A	0.945592	0.672665	0.951077	0.056*	
H29B	0.797407	0.702220	0.996161	0.056*	
H29C	0.829942	0.810909	0.923502	0.056*	
C6	0.8126 (3)	1.1179 (3)	0.49584 (11)	0.0323 (5)	
H6	0.891433	1.162922	0.464320	0.039*	
C31	0.7218 (3)	0.8771 (3)	0.76655 (14)	0.0431 (6)	
H31A	0.639953	0.820185	0.750234	0.065*	
H31B	0.744630	0.971775	0.733154	0.065*	
H31C	0.687716	0.903397	0.809501	0.065*	
C32	0.9431 (3)	0.7677 (3)	0.70892 (13)	0.0485 (7)	
H32A	1.035816	0.703364	0.716090	0.073*	
H32B	0.971724	0.871329	0.684381	0.073*	
H32C	0.870715	0.722351	0.682492	0.073*	

### Atomic displacement parameters (Å^2^)

**Table:**

	*U*^11^	*U*^22^	*U*^33^	*U*^12^	*U*^13^	*U*^23^
S1	0.0187 (2)	0.0157 (2)	0.0222 (3)	−0.00142 (18)	0.00128 (19)	−0.00441 (18)
Si	0.0192 (3)	0.0161 (3)	0.0241 (3)	−0.0013 (2)	0.0001 (2)	−0.0050 (2)
O1	0.0210 (7)	0.0205 (7)	0.0231 (8)	0.0017 (5)	−0.0009 (6)	−0.0061 (6)
O2	0.0307 (8)	0.0274 (8)	0.0293 (8)	0.0035 (6)	0.0014 (7)	−0.0099 (6)
O3	0.0310 (8)	0.0248 (8)	0.0324 (9)	0.0012 (6)	0.0034 (7)	−0.0072 (7)
N1	0.0217 (8)	0.0165 (8)	0.0285 (10)	−0.0009 (7)	0.0028 (7)	−0.0043 (7)
N3	0.0216 (8)	0.0197 (8)	0.0248 (9)	−0.0007 (7)	−0.0004 (7)	−0.0048 (7)
N2	0.0200 (8)	0.0174 (8)	0.0284 (10)	−0.0009 (7)	−0.0009 (7)	−0.0079 (7)
C17	0.0173 (9)	0.0145 (9)	0.0250 (11)	0.0001 (7)	0.0016 (8)	−0.0028 (8)
C11	0.0200 (10)	0.0218 (10)	0.0258 (11)	−0.0034 (8)	0.0006 (8)	−0.0125 (8)
C4	0.0222 (10)	0.0243 (10)	0.0241 (11)	−0.0004 (8)	−0.0059 (8)	−0.0020 (8)
C30	0.0274 (11)	0.0192 (10)	0.0296 (12)	−0.0060 (8)	−0.0017 (9)	−0.0079 (9)
C16	0.0242 (11)	0.0237 (10)	0.0300 (12)	0.0011 (8)	−0.0012 (9)	−0.0111 (9)
C9	0.0265 (11)	0.0247 (10)	0.0229 (11)	−0.0026 (9)	−0.0067 (9)	−0.0040 (9)
C20	0.0204 (10)	0.0188 (10)	0.0344 (13)	−0.0001 (8)	0.0052 (9)	−0.0015 (9)
C18	0.0232 (10)	0.0189 (9)	0.0264 (11)	−0.0016 (8)	0.0008 (8)	−0.0056 (8)
C3	0.0213 (10)	0.0231 (10)	0.0230 (11)	0.0008 (8)	−0.0030 (8)	−0.0035 (9)
C21	0.0223 (10)	0.0193 (10)	0.0395 (13)	−0.0033 (8)	−0.0017 (9)	−0.0078 (9)
C10	0.0249 (10)	0.0228 (10)	0.0235 (11)	0.0011 (8)	−0.0066 (9)	−0.0061 (8)
C12	0.0226 (10)	0.0226 (10)	0.0285 (12)	−0.0012 (8)	0.0002 (9)	−0.0097 (9)
C22	0.0260 (11)	0.0230 (10)	0.0259 (11)	−0.0016 (8)	0.0009 (9)	−0.0076 (9)
C1	0.0188 (10)	0.0239 (10)	0.0264 (11)	0.0010 (8)	0.0025 (8)	−0.0054 (9)
C19	0.0281 (11)	0.0229 (10)	0.0248 (11)	0.0018 (9)	0.0037 (9)	−0.0032 (9)
C24	0.0218 (10)	0.0239 (10)	0.0283 (12)	0.0000 (8)	−0.0008 (9)	−0.0076 (9)
C2	0.0194 (10)	0.0230 (10)	0.0287 (12)	−0.0034 (8)	0.0010 (8)	−0.0042 (9)
C13	0.0311 (11)	0.0255 (11)	0.0284 (12)	−0.0091 (9)	0.0047 (9)	−0.0100 (9)
C27	0.0231 (10)	0.0251 (10)	0.0265 (11)	0.0004 (8)	−0.0001 (8)	−0.0074 (9)
C8	0.0402 (13)	0.0267 (11)	0.0292 (12)	−0.0027 (10)	−0.0073 (10)	−0.0068 (9)
C28	0.0256 (11)	0.0321 (11)	0.0329 (13)	−0.0004 (9)	0.0039 (9)	−0.0131 (10)
C25	0.0263 (11)	0.0248 (11)	0.0376 (13)	0.0044 (9)	−0.0068 (9)	−0.0055 (10)
C5	0.0242 (11)	0.0304 (11)	0.0274 (12)	−0.0012 (9)	−0.0035 (9)	0.0008 (9)
C15	0.0211 (11)	0.0327 (12)	0.0409 (14)	0.0001 (9)	0.0015 (9)	−0.0183 (10)
C14	0.0241 (11)	0.0350 (12)	0.0365 (13)	−0.0104 (9)	0.0085 (9)	−0.0193 (10)
C7	0.0419 (13)	0.0284 (11)	0.0319 (13)	−0.0137 (10)	−0.0111 (11)	−0.0014 (10)
C23	0.0301 (12)	0.0289 (12)	0.0441 (15)	−0.0060 (10)	0.0086 (10)	0.0008 (10)
C26	0.0219 (11)	0.0357 (12)	0.0435 (14)	0.0002 (9)	−0.0011 (10)	−0.0111 (11)
C29	0.0293 (12)	0.0521 (15)	0.0368 (14)	−0.0035 (11)	0.0009 (10)	−0.0254 (12)
C6	0.0297 (12)	0.0353 (12)	0.0290 (12)	−0.0102 (10)	−0.0053 (10)	0.0011 (10)
C31	0.0372 (13)	0.0207 (11)	0.0650 (18)	−0.0019 (10)	−0.0084 (12)	0.0075 (11)
C32	0.0789 (19)	0.0266 (12)	0.0378 (15)	−0.0098 (13)	0.0190 (14)	−0.0045 (11)

### Geometric parameters (Å, º)

**Table:**

S1—N1	1.5139 (16)	C19—H19	0.9500
S1—N2	1.4838 (16)	C24—H24	1.0000
S1—O1	1.6257 (14)	C24—C25	1.537 (3)
S1—C17	1.7718 (19)	C24—C26	1.539 (3)
Si—N2	1.7240 (17)	C2—H2A	0.9900
Si—C30	1.881 (2)	C2—H2B	0.9900
Si—C24	1.882 (2)	C13—H13	0.9500
Si—C27	1.894 (2)	C13—C14	1.383 (3)
O1—C1	1.452 (2)	C27—H27	1.0000
O2—C10	1.211 (2)	C27—C28	1.539 (3)
O3—C3	1.211 (2)	C27—C29	1.539 (3)
N1—C11	1.412 (2)	C8—H8	0.9500
N3—C3	1.396 (2)	C8—C7	1.394 (3)
N3—C10	1.398 (2)	C28—H28A	0.9800
N3—C2	1.459 (2)	C28—H28B	0.9800
C17—C18	1.374 (3)	C28—H28C	0.9800
C17—C22	1.392 (3)	C25—H25A	0.9800
C11—C16	1.394 (3)	C25—H25B	0.9800
C11—C12	1.394 (3)	C25—H25C	0.9800
C4—C9	1.388 (3)	C5—H5	0.9500
C4—C3	1.489 (3)	C5—C6	1.392 (3)
C4—C5	1.380 (3)	C15—H15	0.9500
C30—H30	1.0000	C15—C14	1.386 (3)
C30—C31	1.531 (3)	C14—H14	0.9500
C30—C32	1.525 (3)	C7—H7	0.9500
C16—H16	0.9500	C7—C6	1.387 (3)
C16—C15	1.389 (3)	C23—H23A	0.9800
C9—C10	1.486 (3)	C23—H23B	0.9800
C9—C8	1.385 (3)	C23—H23C	0.9800
C20—C21	1.393 (3)	C26—H26A	0.9800
C20—C19	1.392 (3)	C26—H26B	0.9800
C20—C23	1.507 (3)	C26—H26C	0.9800
C18—H18	0.9500	C29—H29A	0.9800
C18—C19	1.389 (3)	C29—H29B	0.9800
C21—H21	0.9500	C29—H29C	0.9800
C21—C22	1.387 (3)	C6—H6	0.9500
C12—H12	0.9500	C31—H31A	0.9800
C12—C13	1.387 (3)	C31—H31B	0.9800
C22—H22	0.9500	C31—H31C	0.9800
C1—H1A	0.9900	C32—H32A	0.9800
C1—H1B	0.9900	C32—H32B	0.9800
C1—C2	1.505 (3)	C32—H32C	0.9800
			
O1—S1—C17	101.13 (8)	N3—C2—C1	113.82 (17)
N1—S1—O1	105.93 (8)	N3—C2—H2A	108.8
N1—S1—C17	101.98 (9)	N3—C2—H2B	108.8
N2—S1—O1	107.27 (8)	C1—C2—H2A	108.8
N2—S1—N1	126.60 (9)	C1—C2—H2B	108.8
N2—S1—C17	111.06 (9)	H2A—C2—H2B	107.7
N2—Si—C30	107.57 (9)	C12—C13—H13	119.6
N2—Si—C24	110.09 (9)	C14—C13—C12	120.8 (2)
S1—N2—Si	142.24 (11)	C14—C13—H13	119.6
N2—Si—C27	105.99 (9)	Si—C27—H27	106.6
C30—Si—C24	110.30 (9)	C28—C27—Si	114.45 (15)
C30—Si—C27	111.23 (9)	C28—C27—H27	106.6
C24—Si—C27	111.50 (9)	C29—C27—Si	112.48 (14)
C1—O1—S1	118.87 (12)	C29—C27—H27	106.6
C11—N1—S1	125.44 (14)	C29—C27—C28	109.50 (17)
C3—N3—C10	112.05 (16)	C9—C8—H8	121.6
C3—N3—C2	123.98 (16)	C9—C8—C7	116.8 (2)
C10—N3—C2	122.95 (16)	C7—C8—H8	121.6
C18—C17—S1	119.20 (15)	C27—C28—H28A	109.5
C18—C17—C22	120.93 (18)	C27—C28—H28B	109.5
C22—C17—S1	119.81 (15)	C27—C28—H28C	109.5
C16—C11—N1	116.63 (18)	H28A—C28—H28B	109.5
C12—C11—N1	124.22 (17)	H28A—C28—H28C	109.5
C12—C11—C16	119.04 (19)	H28B—C28—H28C	109.5
C9—C4—C3	108.23 (18)	C24—C25—H25A	109.5
C5—C4—C9	121.8 (2)	C24—C25—H25B	109.5
C5—C4—C3	129.93 (19)	C24—C25—H25C	109.5
Si—C30—H30	107.8	H25A—C25—H25B	109.5
C31—C30—Si	111.13 (14)	H25A—C25—H25C	109.5
C31—C30—H30	107.8	H25B—C25—H25C	109.5
C32—C30—Si	112.26 (14)	C4—C5—H5	121.5
C32—C30—H30	107.8	C4—C5—C6	117.0 (2)
C32—C30—C31	110.0 (2)	C6—C5—H5	121.5
C11—C16—H16	119.9	C16—C15—H15	119.7
C15—C16—C11	120.2 (2)	C14—C15—C16	120.6 (2)
C15—C16—H16	119.9	C14—C15—H15	119.7
C4—C9—C10	108.12 (18)	C13—C14—C15	119.2 (2)
C8—C9—C4	121.5 (2)	C13—C14—H14	120.4
C8—C9—C10	130.3 (2)	C15—C14—H14	120.4
C21—C20—C23	121.1 (2)	C8—C7—H7	119.2
C19—C20—C21	118.59 (18)	C6—C7—C8	121.6 (2)
C19—C20—C23	120.3 (2)	C6—C7—H7	119.2
C17—C18—H18	120.2	C20—C23—H23A	109.5
C17—C18—C19	119.65 (19)	C20—C23—H23B	109.5
C19—C18—H18	120.2	C20—C23—H23C	109.5
O3—C3—N3	125.02 (18)	H23A—C23—H23B	109.5
O3—C3—C4	129.27 (19)	H23A—C23—H23C	109.5
N3—C3—C4	105.70 (16)	H23B—C23—H23C	109.5
C20—C21—H21	119.4	C24—C26—H26A	109.5
C22—C21—C20	121.12 (19)	C24—C26—H26B	109.5
C22—C21—H21	119.4	C24—C26—H26C	109.5
O2—C10—N3	124.20 (19)	H26A—C26—H26B	109.5
O2—C10—C9	129.97 (19)	H26A—C26—H26C	109.5
N3—C10—C9	105.82 (17)	H26B—C26—H26C	109.5
C11—C12—H12	119.9	C27—C29—H29A	109.5
C13—C12—C11	120.15 (19)	C27—C29—H29B	109.5
C13—C12—H12	119.9	C27—C29—H29C	109.5
C17—C22—H22	120.5	H29A—C29—H29B	109.5
C21—C22—C17	118.94 (19)	H29A—C29—H29C	109.5
C21—C22—H22	120.5	H29B—C29—H29C	109.5
O1—C1—H1A	110.2	C5—C6—H6	119.4
O1—C1—H1B	110.2	C7—C6—C5	121.2 (2)
O1—C1—C2	107.48 (15)	C7—C6—H6	119.4
H1A—C1—H1B	108.5	C30—C31—H31A	109.5
C2—C1—H1A	110.2	C30—C31—H31B	109.5
C2—C1—H1B	110.2	C30—C31—H31C	109.5
C20—C19—H19	119.6	H31A—C31—H31B	109.5
C18—C19—C20	120.8 (2)	H31A—C31—H31C	109.5
C18—C19—H19	119.6	H31B—C31—H31C	109.5
Si—C24—H24	106.0	C30—C32—H32A	109.5
C25—C24—Si	113.53 (14)	C30—C32—H32B	109.5
C25—C24—H24	106.0	C30—C32—H32C	109.5
C25—C24—C26	110.03 (18)	H32A—C32—H32B	109.5
C26—C24—Si	114.45 (14)	H32A—C32—H32C	109.5
C26—C24—H24	106.0	H32B—C32—H32C	109.5
			
S1—O1—C1—C2	154.80 (13)	C9—C4—C3—N3	−1.7 (2)
S1—N1—C11—C16	153.82 (16)	C9—C4—C5—C6	−0.5 (3)
S1—N1—C11—C12	−30.1 (3)	C9—C8—C7—C6	−0.7 (3)
S1—C17—C18—C19	−177.16 (14)	C20—C21—C22—C17	−0.5 (3)
S1—C17—C22—C21	177.63 (15)	C18—C17—C22—C21	0.5 (3)
O1—S1—N1—C11	71.56 (18)	C3—N3—C10—O2	176.21 (19)
O1—S1—N2—Si	−143.37 (17)	C3—N3—C10—C9	−2.9 (2)
O1—S1—C17—C18	−138.15 (15)	C3—N3—C2—C1	105.9 (2)
O1—S1—C17—C22	44.69 (16)	C3—C4—C9—C10	0.0 (2)
O1—C1—C2—N3	−74.8 (2)	C3—C4—C9—C8	−177.80 (19)
N1—S1—O1—C1	176.34 (13)	C3—C4—C5—C6	177.5 (2)
N1—S1—N2—Si	−17.3 (2)	C21—C20—C19—C18	0.4 (3)
N1—S1—C17—C18	112.72 (16)	C10—N3—C3—O3	−176.40 (19)
N1—S1—C17—C22	−64.45 (17)	C10—N3—C3—C4	2.9 (2)
N1—C11—C16—C15	174.52 (18)	C10—N3—C2—C1	−86.5 (2)
N1—C11—C12—C13	−174.88 (18)	C10—C9—C8—C7	−177.2 (2)
N2—S1—O1—C1	−46.09 (15)	C12—C11—C16—C15	−1.8 (3)
N2—S1—N1—C11	−55.1 (2)	C12—C13—C14—C15	−1.8 (3)
N2—S1—C17—C18	−24.57 (18)	C22—C17—C18—C19	0.0 (3)
N2—S1—C17—C22	158.26 (15)	C19—C20—C21—C22	0.1 (3)
N2—Si—C30—C31	55.01 (18)	C24—Si—N2—S1	−0.8 (2)
N2—Si—C30—C32	−68.60 (19)	C24—Si—C30—C31	175.10 (16)
N2—Si—C24—C25	−61.52 (17)	C24—Si—C30—C32	51.5 (2)
N2—Si—C24—C26	171.01 (15)	C24—Si—C27—C28	−157.85 (14)
N2—Si—C27—C28	−38.05 (17)	C24—Si—C27—C29	76.34 (17)
N2—Si—C27—C29	−163.87 (15)	C2—N3—C3—O3	−7.7 (3)
C17—S1—O1—C1	70.31 (14)	C2—N3—C3—C4	171.58 (17)
C17—S1—N1—C11	176.97 (17)	C2—N3—C10—O2	7.4 (3)
C17—S1—N2—Si	106.97 (18)	C2—N3—C10—C9	−171.74 (17)
C17—C18—C19—C20	−0.5 (3)	C27—Si—N2—S1	−121.54 (18)
C11—C16—C15—C14	0.6 (3)	C27—Si—C30—C31	−60.64 (18)
C11—C12—C13—C14	0.7 (3)	C27—Si—C30—C32	175.74 (17)
C4—C9—C10—O2	−177.3 (2)	C27—Si—C24—C25	55.82 (18)
C4—C9—C10—N3	1.7 (2)	C27—Si—C24—C26	−71.65 (18)
C4—C9—C8—C7	0.0 (3)	C8—C9—C10—O2	0.2 (4)
C4—C5—C6—C7	−0.1 (3)	C8—C9—C10—N3	179.2 (2)
C30—Si—N2—S1	119.40 (18)	C8—C7—C6—C5	0.7 (3)
C30—Si—C24—C25	179.92 (15)	C5—C4—C9—C10	178.36 (18)
C30—Si—C24—C26	52.46 (18)	C5—C4—C9—C8	0.6 (3)
C30—Si—C27—C28	78.57 (17)	C5—C4—C3—O3	−0.7 (4)
C30—Si—C27—C29	−47.24 (18)	C5—C4—C3—N3	−179.9 (2)
C16—C11—C12—C13	1.1 (3)	C23—C20—C21—C22	179.82 (19)
C16—C15—C14—C13	1.2 (3)	C23—C20—C19—C18	−179.31 (18)
C9—C4—C3—O3	177.6 (2)		

### Hydrogen-bond geometry (Å, º)

**Table:**

*D*—H···*A*	*D*—H	H···*A*	*D*···*A*	*D*—H···*A*
C15—H15···O2^i^	0.95	2.52	3.429 (3)	160
C22—H22···O2^ii^	0.95	2.64	3.348 (3)	132
C14—H14···O3^iii^	0.95	2.52	3.429 (3)	161
C31—H31*B*···N1^iv^	0.98	2.60	3.361 (3)	135

Symmetry codes: (i) *x*+1, *y*−1, *z*; (ii) *x*, *y*−1, *z*; (iii) −*x*+2, −*y*+1, −*z*+1; (iv) *x*, *y*+1, *z*.
