# Supplementary material for: Synthesis and crystal structure of rac-2-(1,3-dioxo­isoindolin-2-yl)ethyl 4-methyl-N-phenyl-N′-(tri­iso­propyl­sil­yl)benzene­sulfondiimidoate: the first member of a new substance class
Source: Acta Crystallogr E Crystallogr Commun. 2022 Jun 10;78(Pt 7):699–702. doi: 10.1107/S2056989022005904 (PMC9260357; doi:10.1107/S2056989022005904)
Supplement: Supplementary file 6 [file e-78-00699-sup3.pdf]

Supporting Information S1-S3

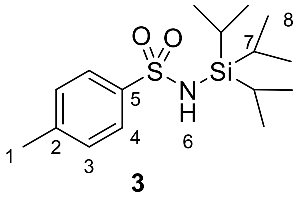

3

S1: molecular scheme of TIPS-protected sulfonamide **3** with atom numbering used in the NMR assignments.

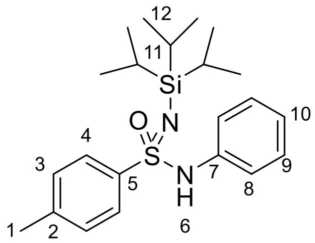

4

S2: molecular scheme of TIPS-protected sulfonimidamide **4** with atom numbering used in the NMR assignments.

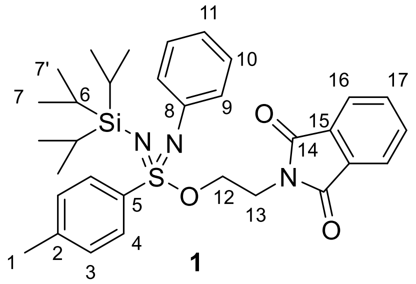

1

S3: molecular scheme of the title compound **1** with atom numbering used in the NMR assignments.
